# Differences in dynamic functional connectivity between musicians and non-musicians during naturalistic music listening

**DOI:** 10.3389/fnins.2025.1649733

**Published:** 2025-10-02

**Authors:** Ruijiao Dai, Petri Toiviainen, Fulvia Francesca Campo, Elvira Brattico

**Affiliations:** ^1^Centre of Excellence in Music, Mind, Body and Brain, Department of Music, Art and Culture Studies, University of Jyväskylä, Jyväskylä, Finland; ^2^Department of Translational Biomedicine and Neuroscience, University of Bari Aldo Moro, Bari, Italy; ^3^Center for Music in the Brain (MIB), Department of Clinical Medicine, Aarhus University and The Royal Academy of Music Aarhus/Aalborg, Aarhus, Denmark; ^4^Department of Education, Psychology, Communication, University of Bari Aldo Moro, Bari, Italy

**Keywords:** naturalistic music listening, dynamic functional connectivity, functional magnetic resonance imaging, musical expertise, neuroplasticity

## Abstract

**Introduction:**

Based on tens of neuroimaging studies and a meta-analysis, we know that music expertise is associated with increases in brain volume and activity in structures related to audition, action, and various cognitive functions. What is less known is how music expertise affects the brain’s functional connectivity during music listening.

**Methods:**

A novel algorithm, Leading Eigenvector Dynamic Analysis, was used to extract the specific dynamic patterns and the corresponding transition process during a naturalistic free-listening paradigm between 18 musicians and 18 non-musicians.

**Results:**

We found a brain state involving the frontal, orbitofrontal, parietal, and anterior cingulate cortex, associated with higher cognitive functions, emotional regulation, and reward, to be more recurrent in musicians during music listening compared to non-musicians. Transition processes revealed heightened engagement in areas linked to auditory processing, somatosensory integration, cognitive function, and emotional regulation in musicians. This enhanced dynamic connectivity might be linked to musicians’ ability to integrate complex auditory stimuli and derive emotional meaning from them. Non-musicians, conversely, showed a tendency to engage more with the default mode network during music listening, possibly in relation to self-relevant mental processes and connection with personal experiences while being absorbed with the music.

**Discussion:**

These findings highlight how music expertise shapes functional connectivity dynamics, musicians enhancing sensorimotor-cognitive integration and non-musicians relying on emotion and personal engagement.

## 1 Introduction

Music stands as a dynamic and universal art form with a significant influence on society, culture, and individual identity. Its ability to evoke emotions, inspire creativity, and build connections makes it a formidable force in our lives. In our modern, fast-paced environment, music functions not only as entertainment but also as a means of relaxation, motivation, and reflection. The process of listening to music is complex, engaging multiple brain regions simultaneously, including those related to somatosensory integration, motor coordination, attention, memory, and emotional processing (Zatorre et al., 2007; Alluri et al., 2017; Vuust et al., 2022). This engagement extends through areas like auditory, visual, motor, memory, and emotional centers. The auditory cortex, which processes sound, becomes active during music listening, helping individuals perceive rhythm, pitch, tone, and melody. Studies have demonstrated that musical expertise, whether gained through long-term or short-term training, enhances the perception and processing of music (Trainor et al., 2003; Kraus and Chandrasekaran, 2010; Tierney et al., 2015; Schneider et al., 2022).

Research indicates significant differences in brain structure and function between musicians and non-musicians. Musicians tend to have larger volumes of gray matter in areas related to auditory processing, motor control, and sensory integration. Studies exploring the influence of music on the brain have demonstrated that musical expertise affects both structural aspects (such as volume and morphology) and functional aspects (including auditory, sensory, emotional, memory, cognitive functions, and neuroplasticity) of the brain (Schlaug, 2001; Schneider et al., 2005; Habib and Besson, 2009; Alluri et al., 2015; Burunat et al., 2015; Palomar-García et al., 2020; Chaddock-Heyman et al., 2021). Criscuolo et al. (2022) reviewed 58 studies on neuroanatomical and functional distinctions between musicians and non-musicians, finding that musicians exhibit increased volume and activity in regions associated with auditory processing, sensorimotor functions, interoception, and emotion.

Specifically, research indicates that musical expertise can improve the auditory system (Trainor et al., 2003; Kraus and Chandrasekaran, 2010; Tierney et al., 2015; Neves et al., 2022) as well as neuroplasticity and cognitive development (Moreno et al., 2011; Miendlarzewska and Trost, 2014; Alves-Pinto et al., 2015; Benz et al., 2016; Saarikivi et al., 2016; Román-Caballero et al., 2022). For instance, Degé et al. (2011) reported enhanced visual and auditory memory in children following two years of participation in a music curriculum. Additionally, other studies have confirmed a correlation between musical expertise and improvements in auditory memory (Cohen et al., 2011). These findings of the auditory cortex undergoing structural changes in response to musical expertise indicate the brain’s remarkable neuroplasticity. Such adaptations contribute to enhancements in a range of cognitive abilities, including memory, attention, language processing, reward systems, and executive functions. Moreover, musicians often have a more developed corpus callosum, the nerve bundle connecting the brain’s two hemispheres, compared to non-musicians (Schlaug et al., 1995; Öztürk et al., n.d.).

Functional connectivity (FC) studies provide in-depth insights into the integrative brain connectome and the impact of musical expertise. Research in this area has shown that musicians typically display enhanced connectivity in brain regions involved in auditory processing, motor skills, memory, and executive functions (Moore et al., 2014; Tanaka and Kirino, 2016; Gujing et al., 2019; Belden et al., 2020; van Vugt et al., 2021). This enhanced connectivity demonstrates the neural adaptability associated with music training and suggests that musical engagement can facilitate more efficient communication between brain regions. For example, the integration of auditory and motor regions is vital for the coordination necessary in playing an instrument, while interactions among areas involved in memory and executive functions can improve creative problem-solving and cognitive flexibility. Burunat et al. (2015) explored the relationship between musical training, callosal anatomy, and interhemispheric functional symmetry during music listening. Their findings revealed that musicians exhibited increased functional symmetry compared to non-musicians.

Additionally, a study by Alluri et al. (2017) examining brain connectivity during naturalistic music listening and the impact of musical expertise found musicians’ enhanced connectivity in limbic, paralimbic, visual, and somatomotor regions of the cerebrum and cerebellum, compared to non-musicians. Alluri et al. (2017) also found that during naturalistic music listening, musicians primarily engaged cerebral and cerebellar sensorimotor regions, while non-musicians predominantly activated areas associated with the default mode network (DMN). Li et al. (2018) reported increased functional connectivity (FC) in both the sensorimotor network and the auditory-motor network in participants undergoing a professional-instructed musical training program.

Despite growing interest in the relationship between musical expertise and brain connectivity, there remains limited understanding of how these connections dynamically reorganize during naturalistic music listening. In this study, we apply Leading Eigenvector Dynamics Analysis (LEiDA; Cabral et al., 2017), a dynamic FC approach, to characterizes the dynamic connectome patterns and transition process between musicians and non-musicians during naturalistic music listening. Unlike traditional dynamic connectivity approaches (e.g., sliding window techniques), it eliminates the need for predefined window lengths and prevents artificial smoothing of rapid neural transitions. Specifically, LEiDA captures instantaneous phase synchrony patterns, providing more precise and sensitive measurements of dynamic functional connectivity.

Drawing on prior findings from this dataset (Alluri et al., 2015, 2017; Burunat et al., 2015; Dai et al., 2024; Brattico et al., 2025) and existing literature, we extend previous analyses by applying LEiDA to characterize dynamic connectivity patterns. We hypothesize that musicians will show distinct dynamic connectivity patterns in auditory-sensorimotor, fronto-limbic and reward-related regions for musicians, reflecting their trained integration of perception, action, and affective evaluation. In contrast, non-musicians are expected to exhibit greater DMN involvement, consistent with their tendency toward internally focused processing during music perception. Furthermore, we anticipate that musicians will demonstrate more frequent transitions between auditory-motor, limbic-frontal, and reward networks, while non-musicians will maintain more sustained DMN-dominant states during music listening.

Although neuroscience research has significantly enhanced our understanding of FC between brain regions during music listening, research focusing on musical expertise remains limited. In this present study, we intend to investigate the dynamic functional connectivity between musicians and non-musicians. For this purpose, we measured the unique patterns and dynamic transition processes during naturalistic music listening by employing the innovative dynamic functional connectivity algorithm LEiDA.

## 2 Materials and methods

### 2.1 Participants

The study protocol was approved by the Coordinating Committee of the Helsinki and Uusimaa Hospital District, Finland, and performed in compliance with the Declaration of Helsinki. The dataset was originally obtained within the large protocol “Tunteet”, which includes fMRI and MEG paradigms datasets, behavioral measurements, and questionnaires. For a general view of the dataset, see (Alluri et al., 2015, 2017; Burunat et al., 2015; Niranjan et al., 2019; Toiviainen et al., 2020; Haumann et al., 2021; Brattico et al., 2025).

In this study, 18 healthy musicians (mean age 28.28 ± 7.79 SD, 9 males) with varying levels of musical expertise and 18 healthy non-musician participants (mean age 29.22 ± 10.66 SD, 8 males) were recruited in this fMRI experiment. The detailed information for musicians and non-musicians is listed in Tables 1, 2, respectively. Informed consent was signed by every participant before the experiment. None of the participants reported any hearing, neurological, or psychological disorders. Participant inclusion criteria were no metal in the body, no tattoo or recent permanent coloring, no pregnancy or breastfeeding, no chronic pharmacological medication, and no claustrophobia. The average training time for the musicians was 15.94 ± 5.68 SD years. The average weekly practice time for musicians was 15.03 ± 12.05 SD hours.

**TABLE 1:** Detailed information about the musicians.

Subject	Age	Hand	Gender	Education	Years training	Weekly practice	Main instrument
1	20	Right	Female	Missing	15	2.5	Cello
2	18	Right	Female	Missing	13	28	Violin, viola
3	20	All	Male	Secondary school	14	25	Piano
4	33	Right	Male	Bachelor’s degree	14	20	Double bass
5	21	Right	Male	Secondary school	12	24.5	Piano
6	25	Right	Female	Missing	20	30	Violin
7	31	Right	Male	Master’s degree	13	15	Mixed, no main
8	44	Right	Male	Music graduate	23	21	Bassoon
9	40	Right	Male	Master’s degree	28	1.5	Cello
10	27	Right	Female	Bachelor’s degree	18	2	Piano
11	34	Right	Female	Master’s degree	17	3.5	Violin
12	35	Right	Female	Bachelor’s degree	20	33	Piano
13	26	Right	Male	Master’s degree	11	4	Piano
14	24	Right	Male	Bachelor’s degree	19	1.5	Trombone
15	22	Right	Female	Bachelor’s degree	7	10	Piano
16	29	Right	Female	Music graduate	24	6	Violin
17	21	Right	Male	Secondary school	7	35	Piano
18	39	Right	Female	Missing	12	8	Piano

**TABLE 2:** Detailed information about the non-musicians.

Subject	Age	Hand	Gender	Education
1	27	Right	Female	Bachelor’s degree
2	19	Right	Male	Secondary school
3	34	Right	Male	PhD
4	23	Right	Female	Secondary school
5	28	Right	Female	Bachelor’s degree
6	21	Right	Female	Secondary school
7	24	Right	Male	Missing
8	26	Right	Male	Missing
9	21	Right	Male	Secondary school
10	33	Right	Female	Master’s degree
11	23	Right	Female	Missing
12	28	Right	Female	Master’s degree
13	52	Right	Male	Master’s degree
14	50	Right	Female	Master’s degree
15	20	Right	Female	Missing
16	29	Left	Male	Missing
17	19	Right	Male	Secondary school
18	49	Right	Female	Master’s degree

### 2.2 Stimuli

During fMRI scanning, participants listened to three naturalistic music pieces: (1) Adios Nonino, composed by the Argentinian composer Astor Piazzolla (1959) (this piece is subsequently simply referred to as “Piazzolla”); (2) Rite of Spring (comprising the first three episodes from Part I: Introduction, The Augurs of Spring: Dances of the Young Girls and Ritual of Abduction) by the Russian born composer Igor Stravinsky (1947); and (3) Stream of Consciousness by Dream Theater (2003). The musical stimuli were delivered using the MR Confon system (Magdeburg, Germany), which is designed for compatibility with MRI environments.

After evaluating three musical pieces and corresponding behavioral and brain responses, Piazzolla is chosen as the musical stimulus in this study due to four key advantages (Alluri et al., 2012; Brattico et al., 2025). First, Piazzolla elicits enhanced neural engagement, driving stronger activation and connection in beauty- and auditory-processing regions during naturalistic listening. Second, Piazzolla demonstrates remarkable structural complexity through sophisticated harmonies, syncopated rhythms, and improvisational elements, which collectively engages both basic auditory processing and higher-order cognitive evaluation systems. Third, behavioral data revealed that Piazzolla received higher familiarity and aesthetic preference ratings compared to the other stimuli. Finally, quantitative analyses of spectral flux and rhythmic regularity confirmed that Piazzolla contains more distinct “aesthetic peaks,” which reliably evoke stronger neural network responses. The duration of Piazzolla is approximately 8 min.

### 2.3 fMRI data acquisition

The fMRI study was conducted using a standard 20-channel head-neck coil with a 3T Siemens MAGNETOM Skyra whole-body scanner at the Advanced Magnetic Imaging (AMI) Center, Aalto University, Finland. A single-shot gradient Echo planar imaging (EPI) sequence (FOV = 192 × 192 mm; 64 × 64 matrix; 33 slices; slice thickness = 4 mm, interslice skip = 0 mm; TE = 32 ms; whole brain, TR = 2 s, flip angle = 75°) was obtained. The 3D T1-weighted structural images (FOV = 256 × 256 mm; 256 × 256 matrix; 176 slices; slice thickness = 1 mm; interslice skip = 0 mm; pulse sequence = Magnetization-Prepared Rapid Gradient-Echo [MPRAGE]) were also acquired for each participant. During fMRI scanning, participants listened to a musical piece while keeping their eyes open and remaining awake, with the volume individually adjusted to a comfortably audible level prior to the session.

### 2.4 Preprocessing

The fMRI data were preprocessed using the MELODIC (Multivariate Exploratory Linear Optimized Decomposition into Independent Components) tool (version 3.15) based on FSL (FMRIB’s Software Library^1^) (Jenkinson et al., 2012) platform (version 6.0.4). First, the raw fMRI data were converted to the Neuro Informatics Technology Initiative (NIfTI) format. The first 4 time points were removed to allow for signal equilibration. Next, slice timing and motion correction were performed to align the acquisition times of different brain slices and mitigate any head movements during the scan, respectively. The fMRI data were then registered from the functional space to the standard Montreal Neurological Institute (MNI) (Evans et al., 1994) space.

Following the initial data preprocessing, Independent Component Analysis (ICA) was applied using the MELODIC tool from FSL. This method decomposed the fMRI data into spatial maps, time courses, and power spectrum, allowing for the removal of noise components associated with human physiological activities–such as respiration, heartbeat, blood flow, and cerebrospinal fluid flow–as well as systematic noise from sources like scanner artifacts, head movements, susceptibility variations, and other complex noise sources (Thomas et al., 2002; Beckmann and Smith, 2004). The ICA components were then manually examined and classified as either signal or artifact components. Subsequently, the FSL function *fsl_regfilt* was performed to filter out the noise components and generate the denoised data. Afterward, the time courses of the BOLD signal were extracted according to Automated Anatomical Labeling (AAL) parcellation into *N* = 90 brain regions. A second-order Butterworth bandpass with a range of 0.02 to 0.1 Hz was applied to the time courses of the BOLD signal.

### 2.5 Leading eigenvector dynamics analysis (LEiDA)

LEiDA (Cabral et al., 2017) (the toolbox can be found in the GitHub repository of Joana Cabral)^2^ was applied in this study to capture the dominant FC pattern of BOLD signals. First, the phase coherence of BOLD signals, which was estimated by the Hilbert transform, was calculated to obtain a time-resolved dynamic FC (dFC) matrix. To calculate the phase coherence, a signal *x* (*t*) can be expressed as *x* (*t*) = *a*(*t*)*cos* [φ(*t*)], where *a*(*t*) represents the instantaneous envelope and φ(*t*) represents the instantaneous phase. Then, the dFC matrix, *dFC*(*n*, *p*, *t*), which represents the phase coherence between brain regions *n* and *p* at each time *t* was obtained by the following equation:


d⁢F⁢C⁢(n,p,t)=c⁢o⁢s⁢[φ⁢(n,t)-φ⁢(p,t)]

where *n*, *p* = 1, 2,…,*N*, *N* represents the number of brain regions (*N* = 90) and *t* = 1, 2,…,*T*, *T* represents the length of the time courses of the BOLD signal. When the two brain regions, *n* and *p*, have an aligned phase at time *t*, the dFC value is: *dFC* (*n*, *p*, *t*)=1. Conversely, if the two brain regions have an anti-aligned phase at time *t*, the dFC value is: *dFC* (*n*, *p*, *t*) = −1. If the two brain regions are orthogonal, the dFC value is: *dFC* (*n*, *p*, *t*) = 0.

At each time point *t*, the dynamic FC matrix *dFC* (*n*, *p*) is an *N* × *N* symmetric matrix due to the nondirectional nature of phase coherence. Consequently, either the upper or lower triangular section of this matrix captures all the significant characteristics of the dFC states. To further minimize the dimensionality of the dFC matrix, a one-dimensional vector known as the leading eigenvector *V*_1_(*t*) is derived through eigenvalue decomposition. The leading eigenvector encapsulates the predominant FC pattern of each dFC matrix, significantly reducing its dimensionality (from *N*(*N*−1)/2 to *N*) and computational complexity. The outer product $V_{1}\,V_{1}^{T}$ (*N* × *N*) can be used to reconstruct the dominant FC matrix.

Each leading eigenvector *V*_1_ consists of *N* elements, each representing a brain region. The sign (positive or negative) of each element in *V*_1_ denotes whether the associated brain regions belong to the same or different community. For instance, if all elements in *V*_1_ share the same sign, it suggests that the phases of the BOLD signal move in the same direction, indicating that all brain regions are part of the same community. Conversely, if *V*_1_ contains elements with both positive and negative signs, it signifies that the phases of the BOLD signal are diverging, thereby dividing the brain regions into two distinct communities (Newman, 2006; Cabral et al., 2017; Figueroa et al., 2019). The magnitude of each eigenvector element reflects the strength of each brain region’s connectivity within its community (Newman, 2006). Since *V* and −*V* indicate the same relative relationships of phase directions, this study ensures that most elements in each leading eigenvector are negative by multiplying them by −1 if necessary.

### 2.6 Phase locking states

To investigate specific phase locking (PL) states associated with music expertise, the *k*-means clustering algorithm was applied to the leading eigenvectors across all time points and subjects, amounting to 8,568 leading eigenvectors across 238 time points for each of the 36 participants. This method enabled the classification of the leading eigenvectors into *k* clusters, with each cluster representing a recurring PL state. In this study, we conducted clustering analyses with *k* values ranging from 2 to 25. For each *k*, the PL states and the transition probabilities between each of the PL states were examined. The *k*-means clustering results highlighted the most significant differences between musicians and non-musicians, which are detailed in the following sections. To investigate the dynamic pattern of brain states of musicians and non-musicians while listening to music, the probability of occurrence of each PL state between two groups were compared with a *t*-test. To assess the statistical significance of each *t*-test comparison, we performed 10,000 permutation tests, which involved randomly shuffling group labels to create a null distribution and determine whether observed differences were stronger than chance. This non-parametric approach avoids distributional assumptions while strictly controlling for false positives.

## 3 Results

### 3.1 Detection of the recurrent PL states

**FIGURE 1:**
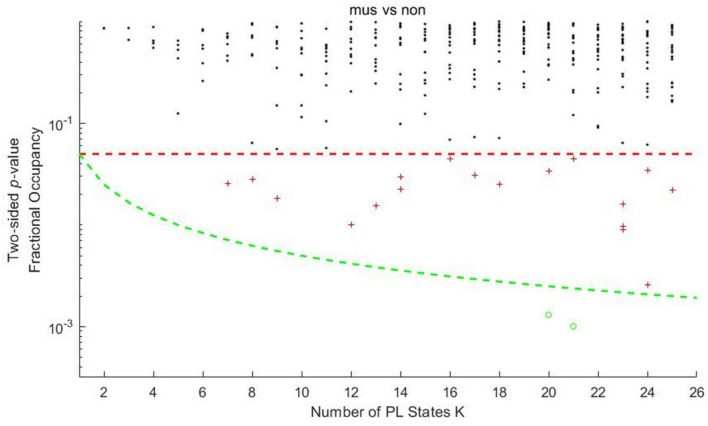
Significance values for the difference of probabilities of the PL states between musicians and non-musicians in each *k*-means clustering model. Each dot represents a *p*-value obtained by the *t*-test calculation of the probability of occurrence of each PL state associated with musicians vs. non-musicians during listening to music as a function of *k*. The red dashed line is the uncorrected threshold of 0.05. The green dashed line is the threshold corrected by the number of clusters (0.05/*k*). The dots (*p*-values) in Figure 1 are marked with different colors depending on their level of significance. The black asterisks represent states with no significant differences in the probability of occurrence between comparisons. Red plus signs represent states that passed the standard threshold (<0.05, uncorrected) but failed the corrected threshold by the number of clusters (0.05/*k*). The green circles represent the states that pass the corrected threshold by the number of clusters.

The probability of occurrences of each PL state during listening to music for musicians and non-musicians was clustered using *k*-means clustering, with *k* ranging from 2 to 25. The results are shown in Figure 1. As we can see in Figure 1, most of the clustering results contain at least one PL state that showed a significant difference between musicians and non-musicians in their probability of occurrence (*p* < 0.05, red plus signs in Figure 1). With *k* equals 20 and 21, two PL states significantly differentiate (*p* < 0.05) when listening to music for musicians vs. non-musicians. In particular, one of the PL states in each model passes the threshold corrected by the number of clusters (*p* < 0.05/*k*, green circles in Figure 1), which reveals the most significant differences between musicians and non-musicians while listening to music. Hence, in this study, we chose the clustering solution *k* = 21 for the following analysis. The PL states across all clustering solutions are visualized in Supplementary Figures 1, 2.

### 3.2 PL states with significant differences

**FIGURE 2:**
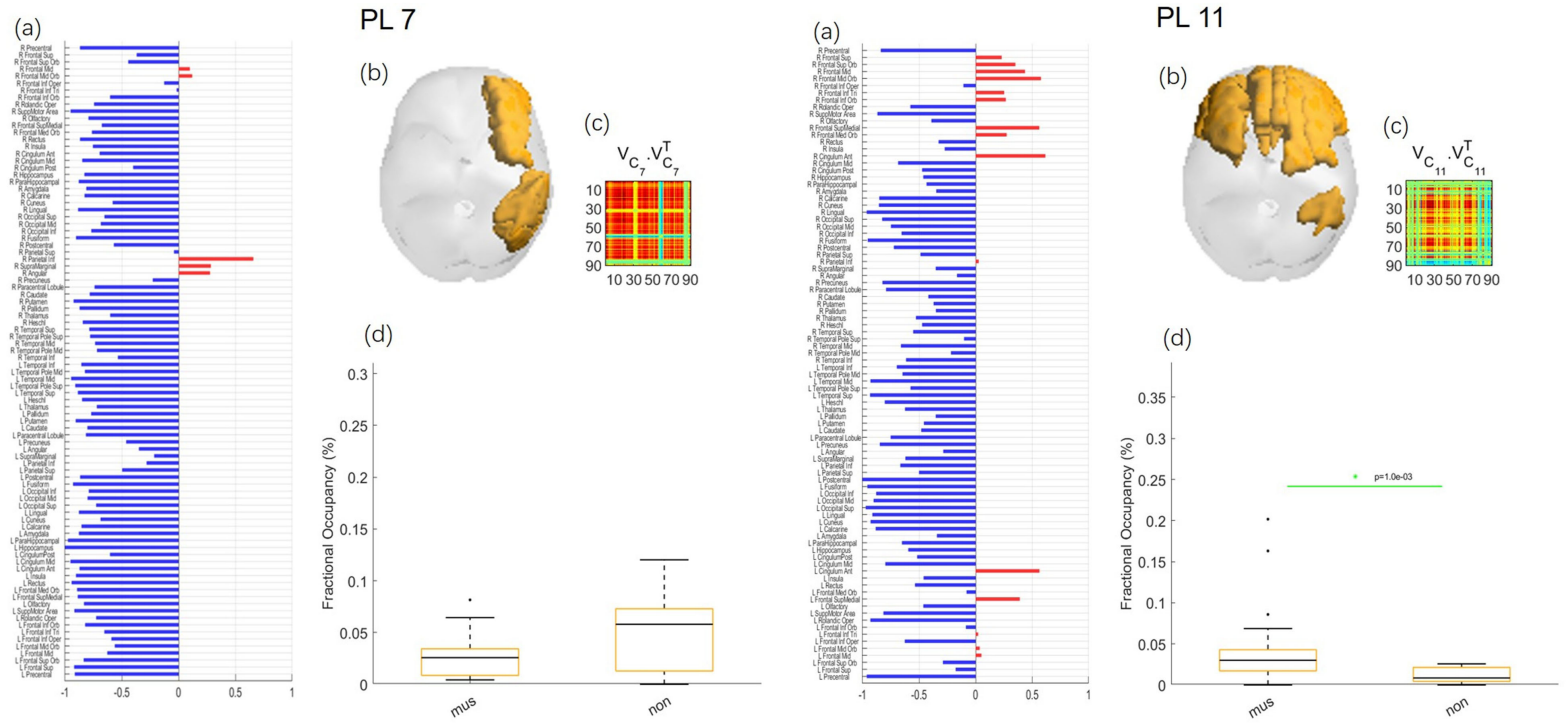
PL states with significant differences between musicians and non-musicians while listening to music. **(a)** PL states represented in vector format. The signs of elements (blue or red) indicate the brain regions belonging to each of the two communities. Regions with the same color represent a connected community, and each eigenvector element’s magnitude shows the strength of each brain region belonging to the PL state. R and L indicate the right or left hemisphere, respectively. **(b)** The transverse view of the PL states. **(c)** The PL states, calculated by $V_{1}\,V_{1}^{T}$, can also be expressed as the outer product of *V*_1_ in matrix format. **(d)** The error bar charts show the probability of occurrence of each PL state between musicians (mus) and non-musicians (non) while listening to music. The green asterisk marks the pattern with the most statistically significant difference (*p* < 0.05/*k*).

With the clustering solution *k* = 21, two PL states were found to differ in their probability between musicians and non-musicians. Both states are displayed in Figure 2.

As we can see in Figure 2, PL state 7 includes the right middle frontal gyrus (MFG), inferior parietal gyrus (IPL), and angular gyrus. The probability of occurrences of the PL state 7 for the musicians (2.84 ± 2.39%) and non-musicians (5.08 ± 3.68%) during listening to music shows significant differences with *p* = 0.0444. PL state 11 includes the right superior frontal gyrus (SFG), bilateral MFG and inferior frontal gyrus (IFG), right orbital frontal gyrus (OFG), bilateral anterior cingulate cortex (ACC), and right IPL. The probability of occurrences of the PL state 11 for the musicians (4.63 ± 5.43%) and non-musicians (1.07 ± 0.85%) during listening to music shows significant differences with *p* = 0.0010.

### 3.3 Transition processes

**FIGURE 3:**
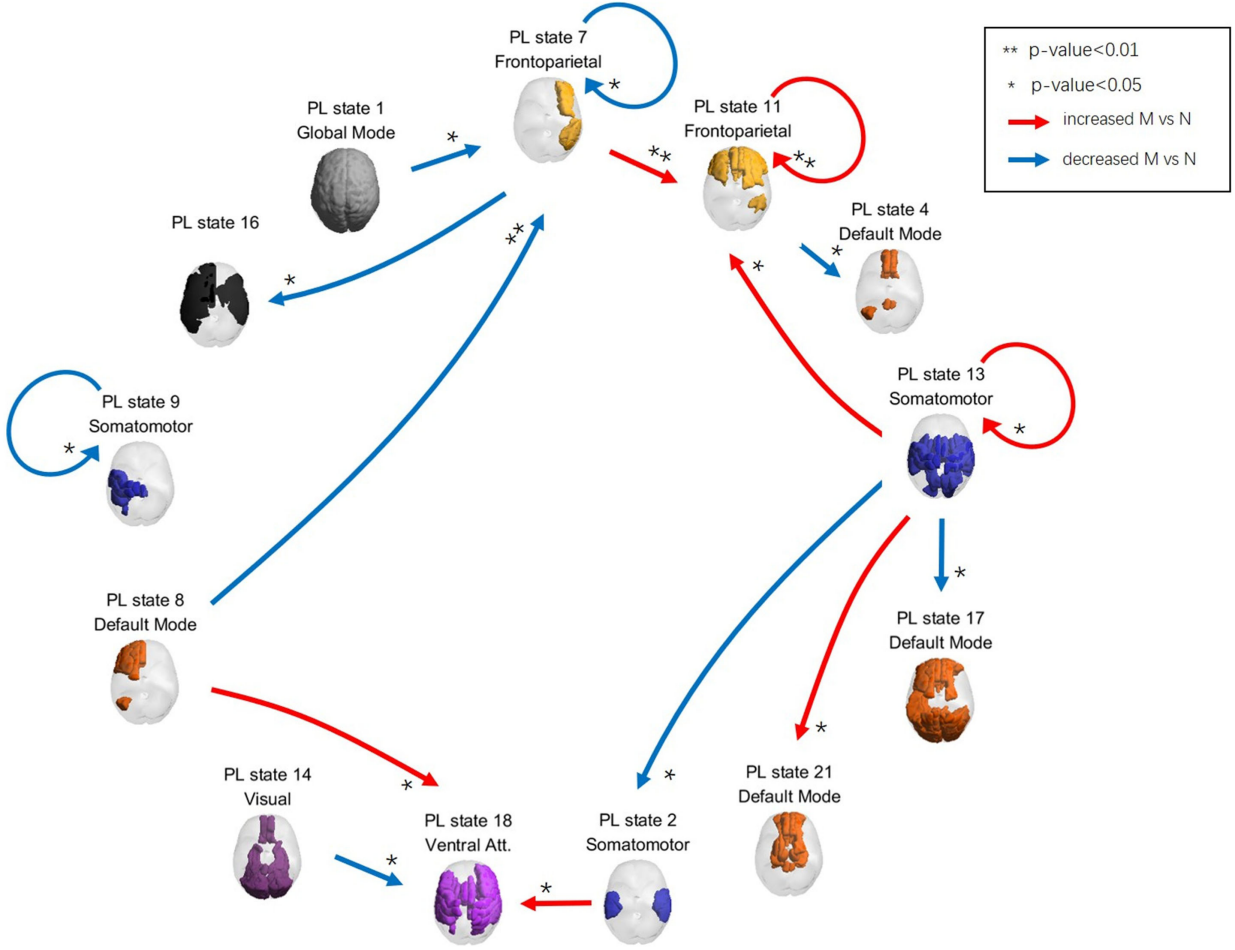
Transition processes for musicians and non-musicians while listening to music. Spatial maps of the PL states and the transition processes with significant probability differences between musicians and non-musicians while listening to music were shown. The red arrows represented transitions with increased probability for musicians compared to non-musicians while listening to music, whereas the blue arrows represented those with decreased probability compared to the non-musicians. M and N in the legend represented the musicians and non-musicians, respectively. Significant differences between musicians and non-musicians during listening to music were indicated with asterisks (*). One asterisk represents the significance threshold *p* < 0.05, while two asterisks represent *p* < 0.01.

The PL state changes from a previous time point to a later one, subsequently referred to as a transition. We calculated the transition probabilities between PL states separately for musicians and non-musicians. Then, we compared the transition probability between musicians and non-musicians with a *t*-test. For each *t*-test, a total of 10000 permutation tests are performed. The transition probabilities with significant differences between the groups are shown in Figure 3.

Figure 3 presents transition processes during music listening for musicians and non-musicians. The diagram illustrates the significant differences in transition probabilities between the two groups: red arrows indicate higher probabilities for musicians, and blue arrows for non-musicians. The canonical networks involved in each PL state during transition processes - including frontoparietal (FPN), salience (SN), DMN, sensorimotor, and limbic networks - are listed in parentheses for their corresponding state.

More specifically, musicians show higher transition probabilities from PL 7 (FPN and DMN) to PL11 (FPN and SN), from PL 8 (DMN) and PL 2 (auditory) to PL 18 (FPN and auditory-motor), from PL 13 (multisensory-limbic) to PL 11 and PL 21 (SN and motor), and recurrent transitions within PL 11 and PL 13 during listening to music compared to non-musicians.

On the other hand, non-musicians show higher transition probabilities from PL 1 (global) and PL 8 to PL 7, from PL 7 to PL 16 (FPN and auditory-limbic), from PL 11 to PL 4 (DMN), from PL 13 to PL 17 (FPN and DMN) and PL 2, from PL 14 (FPN and visual) to PL 18, and recurrent transitions within PL 7 and PL 9 (motor) during listening to music compared to musicians.

## 4 Discussion

In this study, we investigated the dynamic connectome associated with naturalistic music listening among musicians and non-musicians by using LEiDA. This method provided distinct advantages over traditional dynamic connectivity approaches (e.g., sliding window techniques) by eliminating the need for predefined window lengths and avoiding artificial smoothing of rapid neural transitions. Specifically, LEiDA captures instantaneous phase synchrony patterns, providing more precise and sensitive measurements of dynamic functional connectivity. The results revealed that musicians exhibited significantly more frequent occurrences of PL state 11 during music listening, a pattern involving key regions of both the frontoparietal (SFG, MFG, IPL) and salience (IFG, OFC, ACC) networks. These brain regions are known to support higher cognitive functions such as working memory, attention, emotional regulation, reward processing, and auditory information integration - consistent with previous findings by Alluri et al. (2017) and Burunat et al. (2015). This more frequent engagement within frontal areas related to executive functions and emotional processing highlights musicians’ heightened involvement compared to non-musicians.

The specific involvement of the OFC and ACC–brain regions associated with emotion and reward processing (Zatorre and Salimpoor, 2013; Koelsch, 2014; Fasano et al., 2023)–suggests stronger neural correlates of these processes in musicians during music listening. The OFC serves as a pivotal neural hub during music listening, facilitating critical functions such as emotional processing, reward evaluation, and social engagement (Blood and Zatorre, 2001; Menon and Levitin, 2005; Salimpoor et al., 2013). Blood and Zatorre (2001) noted activation of the OFC, ventromedial prefrontal cortex, insula, and ventral striatum during pleasurable musical experiences. Additionally, the OFC is reported to be engaged in music listening, particularly in the context of aesthetic appreciation (Dai et al., 2024; Brattico et al., 2025). Another electroencephalography study showed increased brain signal complexity in the OFC, ACC, auditory cortex, and somatosensory cortex among kindergarten-aged children following 20 days of music training (Carpentier et al., 2016). Our findings also demonstrated that musicians exhibit heightened engagement of the OFC and ACC during music listening compared to non-musicians. The engagement of the OFC and ACC underscores its critical role in processing musical emotions and mediating reward and punishment mechanisms evoked by music, emphasizing its centrality within the neural framework of music perception and cognition.

Moreover, our findings revealed heightened engagement of the frontal lobe, comprising the SFG, MFG, and IFG, in musicians during music listening when compared to non-musicians. The frontal lobe is reported to play critical roles in executive functions such as working memory, attention, and cognitive control, which are essential for processing complex musical structures and maintaining focus during music listening (Popescu et al., 2004; Tabei, 2015; Ding et al., 2019). Studies on musical expertise have demonstrated the critical engagement of the frontal lobe in the neural processing of music (Bangert et al., 2006; Zuk et al., 2014; Chaddock-Heyman et al., 2021). In musicians, these regions are likely more engaged due to their musical expertise, enabling them to decode and interpret intricate auditory patterns more efficiently.

Additionally, we explored the transition processes occurring during music listening, observing switches from one PL state to another. Musicians displayed more frequent transitions involving the auditory and visual cortices, frontal cortex, OFC, and IPL (PL 11 and PL 13) compared to their non-musician counterparts. Notably, PL state 13 overlapped with both the auditory-motor and dorsal attention networks–a pattern aligning with enhanced action-perception coupling in trained musicians (Alluri et al., 2012). Furthermore, we observed increased transition probabilities in brain regions associated with sensory and somatosensory integration, cognitive functions, and emotional awareness. These heightened transition activities may reflect more integrated and flexible neural dynamics in musicians, which could support efficient coordination of sensory, cognitive, and emotional processing. The increased transition probability in regions related to sensory and somatosensory integration resonates with the musicians’ enhanced ability to link auditory stimuli with other sensory modalities. This phenomenon was demonstrated by Proverbio et al. (2015), who found that musicians exhibit enhanced development of audio-visuomotor associations.

We also examined the transition dynamics in non-musicians during music listening, revealing that they engaged sequentially with brain regions including the visual, auditory, frontal, limbic, parietal cortices, the DMN, and the basal ganglia. Notably, the primary transitions occurred from the global (PL 1) and DMN states (PL 8) to the MFG, IPL, and angular gyrus (PL 7) –areas crucial for comprehension, articulatory processing, attention, memory retrieval, and the integration of sensory information. Notably, PL state 8 showed predominant DMN involvement, a network widely associated with self-referential processing and memory retrieval (Gusnard et al., 2001). This suggests that as non-musicians engage with music, they activate regions responsible for interpreting and organizing auditory experiences, reflecting a systematic approach to sensory and cognitive processing. The involvement of these regions highlights the brain’s adaptive capacity to integrate and make sense of complex auditory information, even in the absence of specialized musical training. This underscores the fundamental neural mechanisms that support music perception across different levels of expertise.

Furthermore, a significant transition was observed from the MFG, IPL, and angular gyrus (PL 7) to the auditory cortex, hippocampus, amygdala, and limbic system (PL 16). Additionally, we found increased transition probabilities from the superior temporal gyrus, supplementary motor area, basal ganglia, hippocampus, amygdala, angular gyrus (PL 13) to the auditory state (PL 2) and the MFG, occipital gyrus, inferior temporal gyrus, parietal gyrus, angular gyrus (PL 17). Among these regions, the MFG, IPL, angular gyrus, and hippocampus are integral components of the DMN, which is vital for internal thought processes, emotional regulation, and the overall construction of personal identity. These transitional patterns indicate that non-musicians displayed more frequent shifts within DMN regions during music listening.

The DMN is essential for internal thought processes, emotional regulation, and the construction of personal identity (Gusnard et al., 2001). Previous research has established that DMN-related regions serve as dominant hubs for non-musicians during continuous music listening (Alluri et al., 2017). The dynamic engagement of the DMN we observed in non-musicians could be associated with processes involved in interpreting and internalizing musical experiences, which may underlie their stronger emotional and cognitive responses. While non-musicians may not process musical elements as efficiently as trained musicians, this dynamic engagement with the DMN is fundamental for interpreting and internalizing musical experiences, fostering a deeper emotional, memory, and cognitive connection to the music, ultimately shaping their unique relationship with music. However, it is also important to note that DMN is also active during periods of mind-wandering and reduced external attention (Fox et al., 2015; Poerio et al., 2017). Therefore, the engagement of DMN observed in non-musician could alternatively reflect a state of increased mind-wandering or reduced task-focused attention during music listening. Future studies incorporating real-time behavioral measures (e.g., experience sampling or attention ratings) are needed to differentiate these possibilities and directly link DMN dynamics to specific subjective experiences during music listening.

Unlike static FC studies, LEiDA captures moment-to-moment fluctuations and dynamic transition processes during time-varying measurements such as music listening. Our results demonstrated LEiDA’s consistency with prior findings (Alluri et al., 2015, 2017; Burunat et al., 2015), revealing greater DMN involvement in non-musicians and stronger engagement of auditory-sensorimotor, fronto-limbic, and reward-related regions in musicians. These findings underscore LEiDA’s applicability and robustness. Furthermore, the dynamic FC patterns and transitions between musicians and non-musicians suggest that LEiDA offers a more nuanced and intervention-sensitive representation of brain activity during music listening compared to static FC measures. However, LEiDA relies on *k*-means clustering to identify recurring brain states, where the arbitrary selection of cluster number (*k*) can significantly influence the results. Additionally, the method’s performance is sensitive to preprocessing steps and the chosen brain parcellation scheme, which may introduce variability in the detected dynamic patterns.

Another limitation of this study is the relatively small sample size. We anticipate that increasing the number of participants in future experiments will yield more robust results. Further studies could also include more detailed neurobehavioral assessments of individual preferences, familiarity, or aesthetic ratings, which could provide valuable insights into the subjective experience of music listening. Expanding on this, by incorporating individual behavioral data, the dynamic transition process can be correlated with musical expertise measures–such as years of training and hours of weekly practice–providing quantitative insights into the relationship between music training and brain dynamics. Additionally, the current study included musicians primarily playing the violin, cello, piano, and bassoon. Moving forward, we aim to enhance our understanding by including musicians who perform on a wider variety of instruments, such as the flute, trumpet, and drums. Furthermore, we intend to categorize musicians according to their instrument families–String, Woodwind, Brass, Percussion, and Keyboard–to investigate how different types of instrumentalists engage distinct brain regions during music listening.

In summary, by using LEiDA to measuring the dynamic connectomes and their transition processes, we extended prior findings (Burunat et al., 2015; Alluri et al., 2017) investigating how musical expertise influence brain connectivity during music listening. While these foundational studies established the biological relevance of the observed brain states, our approach provides novel understanding of their temporal coordination and sequential organization. Our findings indicate that musicians exhibit more frequent transitions between distinct brain states, activating regions associated with auditory processing, sensory and somatosensory integration, cognitive functions, memory, and emotional regulation. This enhanced dynamic connectivity reflects their capacity to seamlessly integrate complex auditory stimuli and respond to music with greater emotional depth.

Conversely, non-musicians demonstrated greater DMN engagement compared to musicians. This neural pattern may reflect their heightened reliance on autobiographical and affective processes during music listening, given the DMN’s role in self-referential cognition. The DMN’s integrative function across cognitive, emotional, and mnemonic domains likely contributes to distinct music processing strategies in non-musicians. While the behavioral data regarding the musical familiarity and aesthetic preference was not collected in this study, the observed neurofunctional differences suggest distinct processing strategies may exist between groups during music listening. Future studies incorporating comprehensive behavioral measures could help validate these preliminary findings and elucidate the precise mechanisms underlying group differences in musical information processing.

Overall, these findings demonstrate that musical expertise reorganizes not just static connectivity but the dynamic interactions between large-scale networks. The LEiDA framework proved particularly valuable for capturing these dynamic interactions that traditional analyses might miss. This work advances our understanding of the neural mechanisms differentiating musical expertise, and future studies could combine these methods with behavioral measures to further explore relationships between dynamic connectivity patterns and music perception.

## Data Availability

The raw data supporting the conclusions of this article will be made available by the authors, without undue reservation.

## Footnotes

1
www.fmrib.ox.ac.uk/fsl

2
https://github.com/juanitacabral/LEiDA

## Supplementary material

Supplementary Figure 1 Phase locking states and the corresponding *p*-values between musicians and non-musicians during music listening as a function of *k*. From top to bottom: PL states obtained for each *k* value from 2 to 25 in sagittal view. Above each PL state, the corresponding *p*-value. Red *p*-values: *p* < 0.05; green *p*-values: *p* < 0.05/*k*.

Supplementary Figure 2 Phase locking states and the corresponding *p*-values with significant differences between musicians and non-musicians during music listening as a function of *k*. The figure uses a two-column format, with *k*-values listed on the left side and the corresponding PL states exhibiting statistically significant differences displayed on the right side in each column. Red *p*-values: *p* < 0.05; green *p*-values: *p* < 0.05/*k*. Boxes emphasize PL states that are similar with the most significant differences (states with *p* < 0.05/*k*) across conditions.

## References

[B1] Alluri V. Brattico E. Toiviainen P. Burunat I. Bogert B. Numminen J. (2015). Musical expertise modulates functional connectivity of limbic regions during continuous music listening. *Psychomusicol. Music Mind Brain* 25 443–454. 10.1037/pmu0000124

[B2] Alluri V. Toiviainen P. Burunat I. Kliuchko M. Vuust P. Brattico E. (2017). Connectivity patterns during music listening: Evidence for action-based processing in musicians. *Hum. Brain Mapp.* 38 2955–2970. 10.1002/hbm.23565 28349620 PMC6866725

[B3] Alluri V. Toiviainen P. Jääskeläinen I. P. Glerean E. Sams M. Brattico E. (2012). Large-scale brain networks emerge from dynamic processing of musical timbre, key and rhythm. *NeuroImage* 59 3677–3689. 10.1016/j.neuroimage.2011.11.019 22116038

[B4] Alves-Pinto A. Turova V. Blumenstein T. Thienel A. Wohlschläger A. Lampe R. (2015). fMRI assessment of neuroplasticity in youths with neurodevelopmental-associated motor disorders after piano training. *Eur. J. Paediatr. Neurol.* 19 15–28. 10.1016/j.ejpn.2014.09.002 25305700

[B5] Bangert M. Peschel T. Schlaug G. Rotte M. Drescher D. Hinrichs H. (2006). Shared networks for auditory and motor processing in professional pianists: Evidence from fMRI conjunction. *NeuroImage* 30 917–926. 10.1016/j.neuroimage.2005.10.044 16380270

[B6] Beckmann C. F. Smith S. M. (2004). Probabilistic independent component analysis for functional magnetic resonance imaging. *IEEE Trans. Med. Imag.* 23 137–152. 10.1109/TMI.2003.822821 14964560

[B7] Belden A. Zeng T. Przysinda E. Anteraper S. A. Whitfield-Gabrieli S. Loui P. (2020). Improvising at rest: Differentiating jazz and classical music training with resting state functional connectivity. *NeuroImage* 207:116384. 10.1016/j.neuroimage.2019.116384 31760149

[B8] Benz S. Sellaro R. Hommel B. Colzato L. S. (2016). Music makes the world go round: The impact of musical training on non-musical cognitive functions—a review. *Front. Psychol.* 6:2023. 10.3389/fpsyg.2015.02023 26779111 PMC4703819

[B9] Blood A. J. Zatorre R. J. (2001). Intensely pleasurable responses to music correlate with activity in brain regions implicated in reward and emotion. *Proc. Natl. Acad. Sci. U. S. A.* 98 11818–11823. 10.1073/pnas.191355898 11573015 PMC58814

[B10] Brattico E. Brusa A. Dietz M. Jacobsen T. Fernandes H. M. Gaggero G. (2025). Beauty and the brain – Investigating the neural and musical attributes of beauty during naturalistic music listening. *Neuroscience* 567 308–325. 10.1016/j.neuroscience.2024.12.008 39662529

[B11] Burunat I. Brattico E. Puoliväli T. Ristaniemi T. Sams M. Toiviainen P. (2015). Action in perception: Prominent visuo-motor functional symmetry in musicians during music listening. *PLoS One* 10:e0138238. 10.1371/journal.pone.0138238 26422790 PMC4589413

[B12] Cabral J. Vidaurre D. Marques P. Magalhães R. Silva Moreira P. Miguel Soares J. (2017). Cognitive performance in healthy older adults relates to spontaneous switching between states of functional connectivity during rest. *Sci. Rep.* 7:5135. 10.1038/s41598-017-05425-7 28698644 PMC5506029

[B13] Carpentier S. M. Moreno S. McIntosh A. R. (2016). Short-term music training enhances complex, distributed neural communication during music and linguistic tasks. *J. Cogn. Neurosci.* 28 1603–1612. 10.1162/jocn_a_00988 27243611 PMC5023326

[B14] Chaddock-Heyman L. Loui P. Weng T. B. Weisshappel R. McAuley E. Kramer A. F. (2021). Musical training and brain volume in older adults. *Brain Sci.* 11:50. 10.3390/brainsci11010050 33466337 PMC7824792

[B15] Cohen M. A. Evans K. K. Horowitz T. S. Wolfe J. M. (2011). Auditory and visual memory in musicians and nonmusicians. *Psychon. Bull. Rev.* 18 586–591. 10.3758/s13423-011-0074-0 21374094 PMC3967744

[B16] Criscuolo A. Pando-Naude V. Bonetti L. Vuust P. Brattico E. (2022). An ALE meta-analytic review of musical expertise. *Sci. Rep.* 12:11726. 10.1038/s41598-022-14959-4 35821035 PMC9276732

[B17] Dai R. Toiviainen P. Vuust P. Jacobsen T. Brattico E. (2024). Beauty is in the brain networks of the beholder: An exploratory functional magnetic resonance imaging study. *Psychol. Aesthet. Creat. Arts* 10.1037/aca0000681 [Epub ahead of print].

[B18] Degé F. Wehrum S. Stark R. Schwarzer G. (2011). The influence of two years of school music training in secondary school on visual and auditory memory. *Eur. J. Dev. Psychol.* 8 608–623. 10.1080/17405629.2011.590668

[B19] Ding Y. Zhang Y. Zhou W. Ling Z. Huang J. Hong B. (2019). Neural correlates of music listening and recall in the human brain. *J. Neurosci.* 39 8112–8123. 10.1523/JNEUROSCI.1468-18.2019 31501297 PMC6786812

[B20] Evans A. C. Kamber M. Collins D. L. MacDonald D. (1994). “An MRI-Based probabilistic atlas of neuroanatomy,” in *Magnetic resonance scanning and epilepsy*, eds Shorvon S. D. Fish D. R. Andermann F. Bydder G. M. Stefan H. (Boston, MA: Springer US), 263–274. 10.1007/978-1-4615-2546-2_48

[B21] Fasano M. C. Cabral J. Stevner A. Vuust P. Cantou P. Brattico E. (2023). The early adolescent brain on music: Analysis of functional dynamics reveals engagement of orbitofrontal cortex reward system. *Hum. Brain Mapp.* 44 429–446. 10.1002/hbm.26060 36069619 PMC9842905

[B22] Figueroa C. A. Cabral J. Mocking R. J. T. Rapuano K. M. van Hartevelt T. J. Deco G. (2019). Altered ability to access a clinically relevant control network in patients remitted from major depressive disorder. *Hum. Brain Mapp.* 40 2771–2786. 10.1002/hbm.24559 30864248 PMC6865599

[B23] Fox K. C. Spreng R. N. Ellamil M. Andrews-Hanna J. R. Christoff K. (2015). The wandering brain: Meta-analysis of functional neuroimaging studies of mind-wandering and related spontaneous thought processes. *Neuroimage* 111 611–621. 10.1016/j.neuroimage.2015.02.039 25725466

[B24] Gujing L. Hui H. Xin L. Lirong Z. Yutong Y. Guofeng Y. (2019). Increased insular connectivity and enhanced empathic ability associated with dance/music training. *Neural Plast* 2019:9693109. 10.1155/2019/9693109 31198419 PMC6526550

[B25] Gusnard D. A. Akbudak E. Shulman G. L. Raichle M. E. (2001). Medial prefrontal cortex and self-referential mental activity: Relation to a default mode of brain function. *Proc. Natl. Acad. Sci. U. S. A.* 98 4259–4264. 10.1073/pnas.071043098 11259662 PMC31213

[B26] Habib M. Besson M. (2009). What do music training and musical experience teach us about brain plasticity? *Music Percept.* 26 279–285. 10.1525/mp.2009.26.3.279

[B27] Haumann N. T. Lumaca M. Kliuchko M. Santacruz J. L. Vuust P. Brattico E. (2021). Extracting human cortical responses to sound onsets and acoustic feature changes in real music, and their relation to event rate. *Brain Res.* 1754:147248. 10.1016/j.brainres.2020.147248 33417893

[B28] Jenkinson M. Beckmann C. F. Behrens T. E. J. Woolrich M. W. Smith S. M. (2012). FSL. *NeuroImage* 62 782–790. 10.1016/j.neuroimage.2011.09.015 21979382

[B29] Koelsch S. (2014). Brain correlates of music-evoked emotions. *Nat. Rev. Neurosci.* 15 170–180. 10.1038/nrn3666 24552785

[B30] Kraus N. Chandrasekaran B. (2010). Music training for the development of auditory skills. *Nat. Rev. Neurosci.* 11 599–605. 10.1038/nrn2882 20648064

[B31] Li Q. Wang X. Wang S. Xie Y. Li X. Xie Y. (2018). Musical training induces functional and structural auditory-motor network plasticity in young adults. *Hum. Brain Mapp.* 39 2098–2110. 10.1002/hbm.23989 29400420 PMC6866316

[B32] Menon V. Levitin D. J. (2005). The rewards of music listening: Response and physiological connectivity of the mesolimbic system. *NeuroImage* 28 175–184. 10.1016/j.neuroimage.2005.05.053 16023376

[B33] Miendlarzewska E. A. Trost W. J. (2014). How musical training affects cognitive development: Rhythm, reward and other modulating variables. *Front. Neurosci.* 7:279. 10.3389/fnins.2013.00279 24672420 PMC3957486

[B34] Moore E. Schaefer R. S. Bastin M. E. Roberts N. Overy K. (2014). Can musical training influence brain connectivity? evidence from diffusion tensor MRI. *Brain Sci.* 4 405–427. 10.3390/brainsci4020405 24961769 PMC4101485

[B35] Moreno S. Bialystok E. Barac R. Schellenberg E. G. Cepeda N. J. Chau T. (2011). Short-Term music training enhances verbal intelligence and executive function. *Psychol. Sci.* 22 1425–1433. 10.1177/0956797611416999 21969312 PMC3449320

[B36] Neves L. Correia A. I. Castro S. L. Martins D. Lima C. F. (2022). Does music training enhance auditory and linguistic processing? A systematic review and meta-analysis of behavioral and brain evidence. *Neurosci. Biobehav. Rev.* 140:104777. 10.1016/j.neubiorev.2022.104777 35843347

[B37] Newman M. E. J. (2006). Finding community structure in networks using the eigenvectors of matrices. *Phys. Rev. E Stat. Nonlin. Soft Matter. Phys.* 74:036104. 10.1103/PhysRevE.74.036104 17025705

[B38] Niranjan D. Toiviainen P. Brattico E. Alluri V. (2019). “Dynamic functional connectivity in the musical brain,” in *Brain informatics*, eds Liang P. Goel V. Shan C. (Cham: Springer International Publishing), 82–91. 10.1007/978-3-030-37078-7_9

[B39] Öztürk A. H. Tasçioglu B. Aktekin M. Kurtoglu Z. Erden I. (n.d.). *Morphometric comparison of the human corpus callosum in professional musicians and non-musicians by using in vivo magnetic resonance imaging.* 11984475

[B40] Palomar-García M. -Á Hernández M. Olcina G. Adrián-Ventura J. Costumero V. Miró-Padilla A. (2020). Auditory and frontal anatomic correlates of pitch discrimination in musicians, non-musicians, and children without musical training. *Brain Struct. Funct.* 225 2735–2744. 10.1007/s00429-020-02151-1 33029708

[B41] Poerio G. L. Sormaz M. Wang H. T. Margulies D. Jefferies E. Smallwood J. (2017). The role of the default mode network in component processes underlying the wandering mind. *Soc. Cogn. Affect. Neurosci.* 12 1047–1062. 10.1093/scan/nsx041 28402561 PMC5490683

[B42] Popescu M. Otsuka A. Ioannides A. A. (2004). Dynamics of brain activity in motor and frontal cortical areas during music listening: A magnetoencephalographic study. *NeuroImage* 21 1622–1638. 10.1016/j.neuroimage.2003.11.002 15050586

[B43] Proverbio A. M. Attardo L. Cozzi M. Zani A. (2015). The effect of musical practice on gesture/sound pairing. *Front. Psychol.* 6:376. 10.3389/fpsyg.2015.00376 25883580 PMC4382982

[B44] Román-Caballero R. Vadillo M. A. Trainor L. J. Lupiáñez J. (2022). Please don’t stop the music: A meta-analysis of the cognitive and academic benefits of instrumental musical training in childhood and adolescence. *Educ. Res. Rev.* 35:100436. 10.1016/j.edurev.2022.100436

[B45] Saarikivi K. Putkinen V. Tervaniemi M. Huotilainen M. (2016). Cognitive flexibility modulates maturation and music-training-related changes in neural sound discrimination. *Eur. J. Neurosci.* 44 1815–1825. 10.1111/ejn.13176 26797826

[B46] Salimpoor V. N. van den Bosch I. Kovacevic N. McIntosh A. R. Dagher A. Zatorre R. J. (2013). Interactions between the nucleus accumbens and auditory cortices predict music reward value. *Science* 340 216–219. 10.1126/science.1231059 23580531

[B47] Schlaug G. (2001). The brain of musicians. *Ann. N. Y. Acad. Sci.* 930 281–299. 10.1111/j.1749-6632.2001.tb05739.x 11458836

[B48] Schlaug G. Jäncke L. Huang Y. Staiger J. F. Steinmetz H. (1995). Increased corpus callosum size in musicians. *Neuropsychologia* 33 1047–1055. 10.1016/0028-3932(95)00045-5 8524453

[B49] Schneider P. Groß C. Bernhofs V. Christiner M. Benner J. Turker S. (2022). Short-term plasticity of neuro-auditory processing induced by musical active listening training. *Ann. N. Y. Acad. Sci.* 1517 176–190. 10.1111/nyas.14899 36114664 PMC9826140

[B50] Schneider P. Sluming V. Roberts N. Scherg M. Goebel R. Specht H. J. (2005). Structural and functional asymmetry of lateral Heschl’s gyrus reflects pitch perception preference. *Nat. Neurosci.* 8 1241–1247. 10.1038/nn1530 16116442

[B51] Tabei K. (2015). Inferior frontal gyrus activation underlies the perception of emotions, while precuneus activation underlies the feeling of emotions during music listening. *Behav. Neurol.* 2015:529043. 10.1155/2015/529043 26504353 PMC4609410

[B52] Tanaka S. Kirino E. (2016). Functional connectivity of the precuneus in female university students with long-term musical training. *Front. Hum. Neurosci.* 10:328. 10.3389/fnhum.2016.00328 27445765 PMC4925677

[B53] Thomas C. G. Harshman R. A. Menon R. S. (2002). Noise reduction in BOLD-Based fMRI using component analysis. *NeuroImage* 17 1521–1537. 10.1006/nimg.2002.1200 12414291

[B54] Tierney A. T. Krizman J. Kraus N. (2015). Music training alters the course of adolescent auditory development. *Proc. Natl. Acad. Sci. U. S. A.* 112 10062–10067. 10.1073/pnas.1505114112 26195739 PMC4538630

[B55] Toiviainen P. Burunat I. Brattico E. Vuust P. Alluri V. (2020). The chronnectome of musical beat. *NeuroImage* 216:116191. 10.1016/j.neuroimage.2019.116191 31525500

[B56] Trainor L. J. Shahin A. Roberts L. E. (2003). Effects of musical training on the auditory cortex in children. *Ann. N. Y. Acad. Sci.* 999 506–513. 10.1196/annals.1284.061 14681174

[B57] van Vugt F. T. Hartmann K. Altenmüller E. Mohammadi B. Margulies D. S. (2021). The impact of early musical training on striatal functional connectivity. *NeuroImage* 238:118251. 10.1016/j.neuroimage.2021.118251 34116147

[B58] Vuust P. Heggli O. A. Friston K. J. Kringelbach M. L. (2022). Music in the brain. *Nat. Rev. Neurosci.* 23 287–305. 10.1038/s41583-022-00578-5 35352057

[B59] Zatorre R. J. Chen J. L. Penhune V. B. (2007). When the brain plays music: Auditory–motor interactions in music perception and production. *Nat. Rev. Neurosci.* 8 547–558. 10.1038/nrn2152 17585307

[B60] Zatorre R. J. Salimpoor V. N. (2013). From perception to pleasure: Music and its neural substrates. *Proc. Natl. Acad. Sci. U. S. A.* 110 10430–10437. 10.1073/pnas.1301228110 23754373 PMC3690607

[B61] Zuk J. Benjamin C. Kenyon A. Gaab N. (2014). Behavioral and neural correlates of executive functioning in musicians and non-musicians. *PLoS One* 9:e99868. 10.1371/journal.pone.0099868 24937544 PMC4061064

