# Supplementary figures and images for: Differences in dynamic functional connectivity between musicians and non-musicians during naturalistic music listening
Source: Front Neurosci. 2025 Oct 2;19:1649733. doi: 10.3389/fnins.2025.1649733 (PMC12528122; doi:10.3389/fnins.2025.1649733)

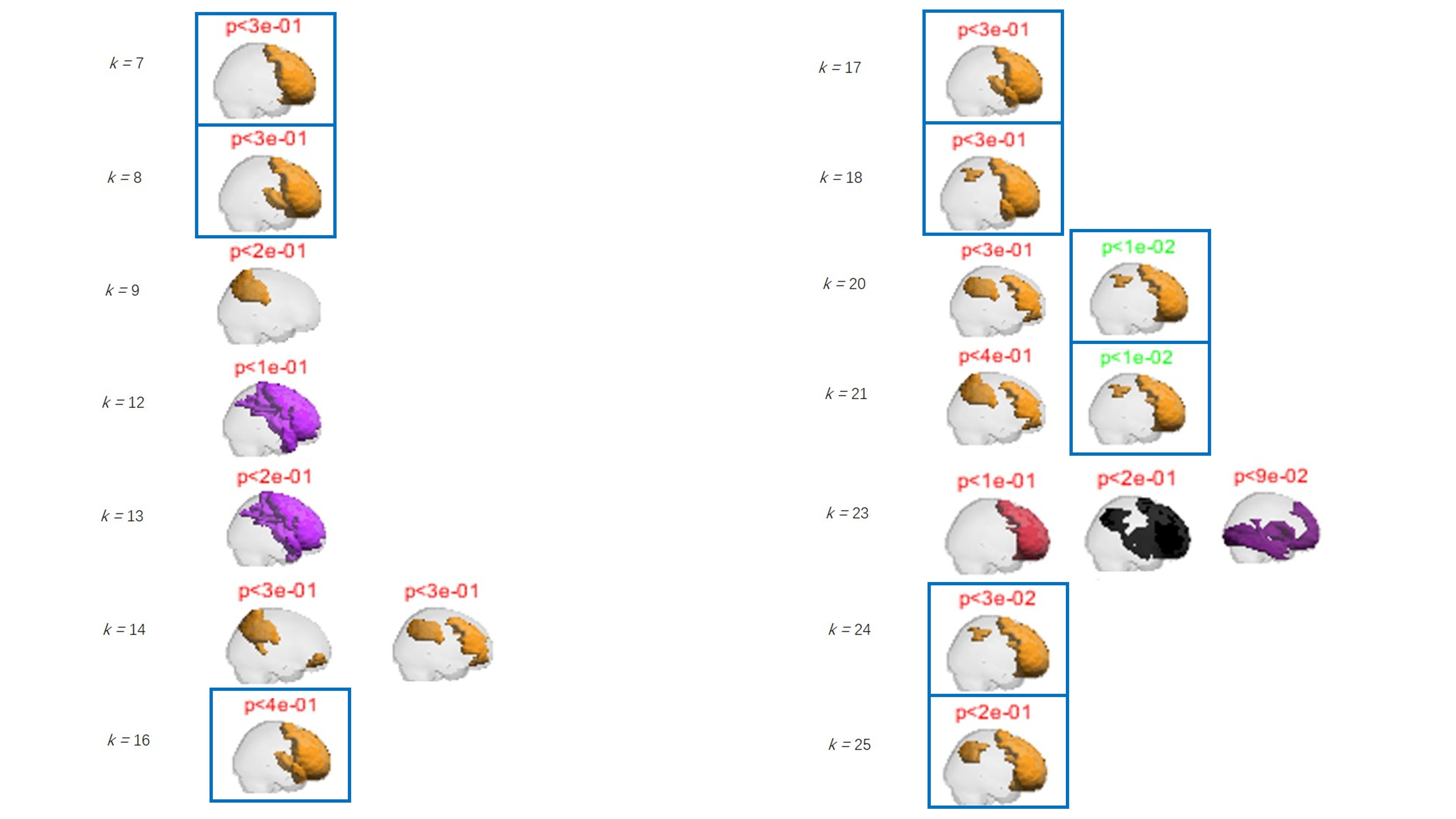

Supplement: Supplementary Figure 1 — Phase locking states and the corresponding p-values between musicians and non-musicians during music listening as a function of k. From top to bottom: PL states obtained for each k value from 2 to 25 in sagittal view. Above each PL state, the corresponding p-value. Red p-values: p < 0.05; green p-values: p < 0.05/k. [file Image_1.JPEG]

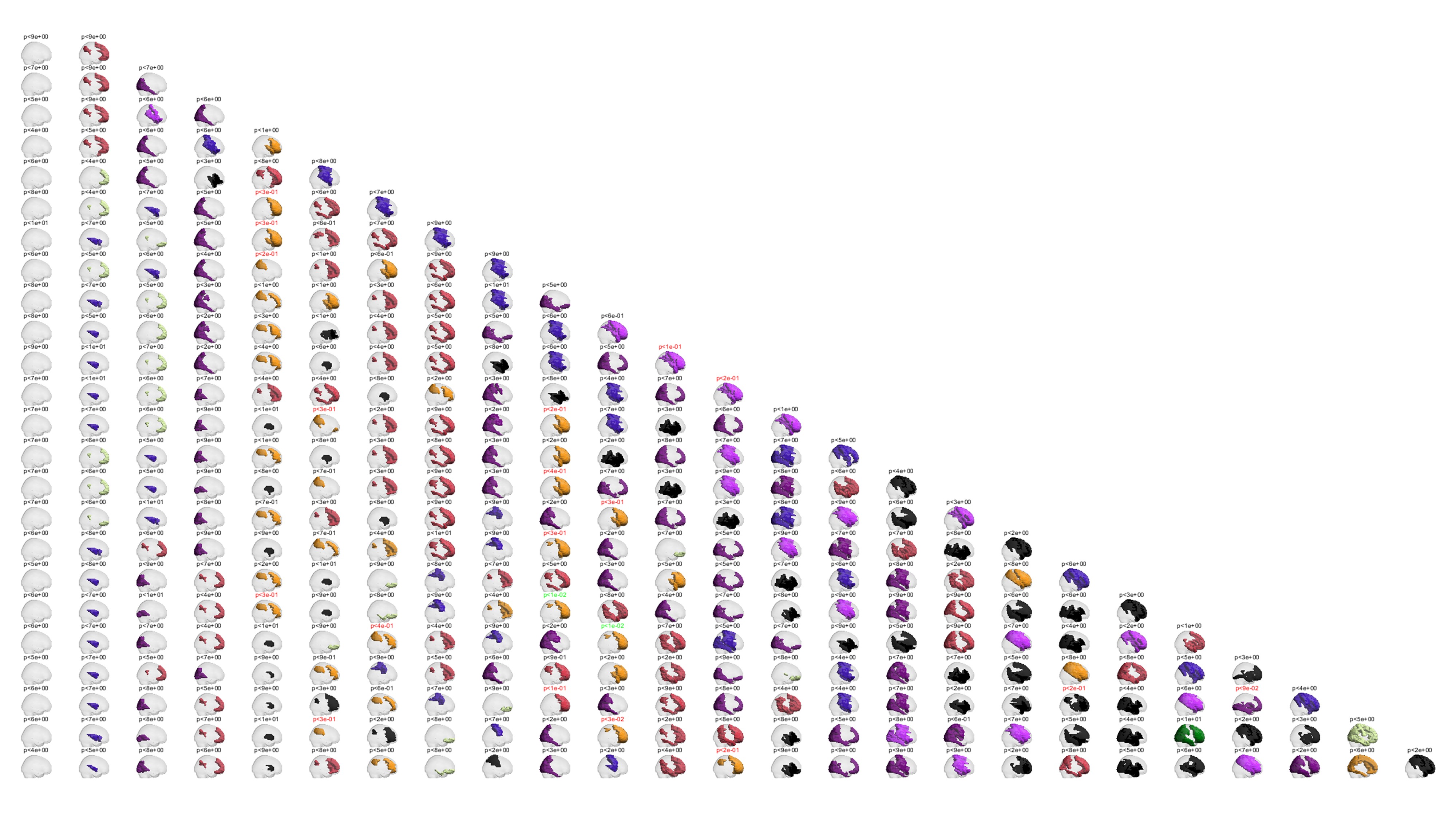

Supplement: Supplementary Figure 2 — Phase locking states and the corresponding p-values with significant differences between musicians and non-musicians during music listening as a function of k. The figure uses a two-column format, with k-values listed on the left side and the corresponding PL states exhibiting statistically significant differences displayed on the right side in each column. Red p-values: p < 0.05; green p-values: p < 0.05/k. Boxes emphasize PL states that are similar with the most significant differences (states with p < 0.05/k) across conditions. [file Image_2.JPEG]
